# Patient Semi-specific Computational Modeling of Electromagnetic Stimulation Applied to Neuroprotective Treatments in Acute Ischemic Stroke

**DOI:** 10.1038/s41598-020-59471-9

**Published:** 2020-02-19

**Authors:** Micol Colella, Francesca Camera, Fioravante Capone, Stefania Setti, Ruggero Cadossi, Vincenzo Di Lazzaro, Francesca Apollonio, Micaela Liberti

**Affiliations:** 1 Department of Information Engineering, Electronics and Telecommunications (DIET), University of Rome “La Sapienza”, Rome, Italy; 2 Unit of Neurology, Neurophysiology, Neurobiology, Department of Medicine, Università Campus Bio-Medico di Roma, Rome, Italy; 3 IGEA Biophysics Laboratory, Carpi, Italy; 4 Present Address: Pervasive Electromagnetics Lab, University of Rome Tor Vergata, Rome, Italy

**Keywords:** Computational models, Stroke, Biomedical engineering

## Abstract

Neuroprotective effects of pulsed electromagnetic fields (PEMFs) have been demonstrated both *in vivo* and *in vitro*. Moreover, preliminary clinical studies have been conducted and suggested PEMFs as a possible alternative therapy to treat acute ischemic stroke. In this work, we show that it’s possible to build-up a patient semi-specific head model, where the 3D reconstruction of the ischemic lesion of the patient under treatment is inserted in the head of the human body model “Duke” (v.1.0, Zurich MedTech AG). The semi-specific model will be used in the randomized, placebo-controlled, double-blind study currently ongoing. Three patients were modelled and simulated, and results showed that each ischemic lesion experiences a magnetic flux density field comparable to the one for which biological effects have been attested. Such a kind of dosimetric analysis reveals a reliable tool to assess the correlation between levels of exposure and the beneficial effect. Thus, once the on-going double blind study is complete it will prove if PEMFs treatment triggers a clinical effect, and we will then be able to characterize a dose-response curve with the methodology arranged in this study.

## Introduction

A cerebral stroke is a sudden insult that occurs after complete or partial blood flow interruption to the brain. Whether this lack of blood flow is due to a restriction or to a full occlusion of arterial vessels supplying the brain, the stroke is said to be ischemic. Ischemic stroke, which accounts for approximately 80% of all strokes, is the third cause of death worldwide and the main cause of chronic, severe adult disabilities^1^. The ischemic lesion consists of a central core, with severely compromise cerebral blood flow (CBF) which leads to immediate neuronal death. The core is surrounded by a rim of ischemic tissue characterized by impaired electrical activity but preserved cellular metabolism, known as the *penumbra*. As an opposite to the core, the penumbra is reversibly damaged. In this case, tissue salvage may be achieved when re-perfusion is established within a 6- to 8-hour period^2^. Even though thrombolysis is nowadays the most widely diffuse therapy to treat strokes, important advances made in the understanding of the pathophysiological mechanisms underlying the evolution of the ischemic penumbra have brought to light the alternative approach of neuroprotection. Neuroprotection aims to antagonize the harmful molecular and cellular events responsible for the ischemic damage, allowing brain cells to survive to the reduced CBF and to stabilize penumbra^1^. Because of this, several studies have suggested the hypothesis that electromagnetic fields exposure may be an interesting approach as a noninvasive treatment with a low impact on daily life^3^. In the early 2000s, extremely low frequency magnetic fields have shown to enhance neurite outgrowth^4^ and to save rat cerebellar granule neurons from apoptosis^5^. More recently, Urnukhsaikhan *et al*. demonstrated a neuroprotective effect of low frequency-pulsed electromagnetic fields (LF-PEMF) in mice after ischemic stroke^6^. Specifically, the authors showed that LF-PEMF exposure increases resistance to apoptosis via activation of the TrkB/Akt/Bad pathway and the upregulation of brain-derived neurotrophic factor (BDNF) expression, while decreasing the level of inflammatory mediators, such as Interleukin 1 beta (IL-1*β*) and Matrix metalloproteinase-9 (MMP9). Moreover, LF-PEMF exposure was able to significantly improve mouse behavior during the recovery process. Pena-Philippides *et al*. studied the effects of PEMF exposure on infarct size and inflammation following distal middle cerebral artery occlusion (dMCAO) in mice^7^. This work demonstrated that PEMFs reduce the size of the ischemic lesion measured by MRI and modify the profile of pro-inflammatory cytokines causing an anti-inflammatory and anti-apoptotic effect. Segal *et al*. studied a very low intensity, low frequency, electromagnetic field treatment (VLIFE) on an *in vivo* stroke rat model^8^. Brain MRI imaging showed a decrease in perilesional edema and lateral ventricle widening in the treated groups. Treated rats showed recovery of sensory-motor functional deficits, as demonstrated by Modified Neurological Severity Score and forelimb placement tests. The authors concluded that VLIFE stimulation promotes neuronal plasticity after stroke, thus improving clinical recovery. In 2002 Varani and co-workers^9^ demonstrated that exposures to low frequency and low energy pulsed electromagnetic fields (PEMFs) induces the upregulation of *A*_2*A*_ adenosine receptors (ARs) in human neutrophils with a magnetic flux density in the range of 1–3.5 mT. A subsequent study, conducted on rat cerebral cortex and cortical neurons, has also confirmed that the effect of PEMFs exposure is selective for *A*_2*A*_ and *A*_3_ ARs^10^. These are fundamental achievements, considering that *A*_2*A*_ and *A*_3_ play a key role in protection against ischemic damage^10,11^. In order to better understand the effect of PEMFs in neuronal cells following hypoxia injury, Vincenzi and co-workers^12^ exposed human neuroblastoma cell line SH-SY5Y and rat pheochromocytoma PC12 cells, cell lines endowed with long term proliferative capacity *in-vitro* and widely accepted as good neuron-like cell models^13,14^. Results showed that PEMFs stimulation significantly reduced hypoxia-induced cell death. The signal used for the *in vitro* experiments by the group of Varani^9,10,12^ is the same that has been used in the study conducted in Grant *et al*. in 1994^15^, where they evaluated potential beneficial effect of PEMFs on rabbit cerebral damage. These results showed attenuated cortical edema, when considering magnetic resonance imaging (MRI), and in a reduced neuronal damage, assessed with histological examination. Besides, the aforementioned signal is also able to produce changes in the excitability of the human cerebral cortex^16^. Moving to human studies, an open-label, one-arm, dose-escalation, exploratory study^17^ was carried out in order to evaluate the safety and tolerability of PEMFs in patients with acute ischemic stroke. The study consisted of a 5-day intervention phase and a 12-month follow-up phase. Within 48 hours from the stroke onset, the six enrolled patients underwent PEMFs treatment daily for 5 consecutive days. Clinical follow up lasted 12 months and brain MRI was performed before, and at the one month mark after the treatment. According to the protocol, the first group of three patients was stimulated for 45 min/day and the second group of three patients for 120 min/day. No adverse events occurred during the treatment and clinical conditions improved in all patients. In particular, lesion size was reduced in one of the patients stimulated for 45 minutes and in all of those stimulated for 120 minutes.

This study opened the pathway to a new second research avenue: an on-going randomized, placebo-controlled, double-blind study (I-NIC)^18^. This clinical trial foresees approximately 124 patients, still in the recruiting phase, and will clarify whether PEMFs represent a novel approach to neuroprotection with the final aim to identify a dose-response curve. This is an essential pre-requistite to apply physical agents as PEMFs as a medical treatment in an *electroceuticals* perspective^19^. The estimation of the electric and magnetic quantities inside the brain tissue that are able to elicit an effect is indeed an unavoidable step to an effective and reliable use of the electric or magnetic stimulation of the brain^20^; one of the most adopted approaches is the use of computational dosimetry^21–33^.

The dosimetric computation can be performed by numerically solving the electromagnetic problem inside realistic brain models, stimulated by simplified or realistic sources models. The quantities calculated are the magnetic flux density field (**B**), electric field (**E**) and current density (**J**) distributions induced in the brain. Indeed, knowledge of **B**, **E**, **J** field distribution is fundamental for evaluating position, level, and extent of the stimulation. Due to the generally high wavelength of the stimulating field with respect to the head size, most of these methods use quasi–static approximation to determine the distribution of the fields induced in the human brain^25–31^.

The present paper aims to identify a methodology for a dose-response curve assessment, suitable to be applied in a large scale clinical study. In particular, it is preparatory for the computational dosimetry that will be carried out on the 124 patients recruited in the multicentric double blind I-NIC study^18^. Therefore, in this paper, a dosimetric study, based on a magneto-quasistatic solution at macroscopic level was performed, using a semi-specific head model of the patients. The novelty of semi-specific approach is to represent the ischemic volume of each patient inserted in the correct position inside a standard human head model. This leads to a faster and reliable procedure suitable for the analysis of a large sample of patients. Moreover, in order to assess a methodology able to identify a threshold and a dose-response curve, we proceeded to obtain a post-treatment model of the ischemia for each patient, that was accurately placed in the semi-specific head model, to compare pre- and post-treatment ischemic volumes. Ratio of the volumes in the head space were correlated to the exposure intensity (**B**) field in order to quantify if, and where, we could observe a significant reduction. Similar calculations have been perfomed also for the **J** field.

Such a methodology is applied here to the reduced sample of the three patients recruited in Capone *et al*.^17^ that underwent magnetic stimulation for 120 minutes (the same treatment duration as the one used in the I-NIC study^18^). Such a pool of data seems to be consistent with the final aim since in both cases, the magnetic source is a single monolateral solenoid placed on the patient head, therefore the dosimetric methodology proposed here will be applicable for the I-NIC study^18^.

As shown in Capone *et al*.^17^, this source application implied a gradient of **B** transverse to the head that decreases with the distance from the solenoid itself. From a dosimetric point of view, this opened the question on the exposure experimented by the different ischemic regions as a function of distance. This is a crucial point in order to be able to identify the minimal exposure threshold to attain the effect.

This study lays the groundwork for a methodology, that once applied to the I-NIC^18^ patients, will be able to identify the stimulation threshold capable of triggering (if any) a neuroprotective effect.

## Results

**Figure 1:**
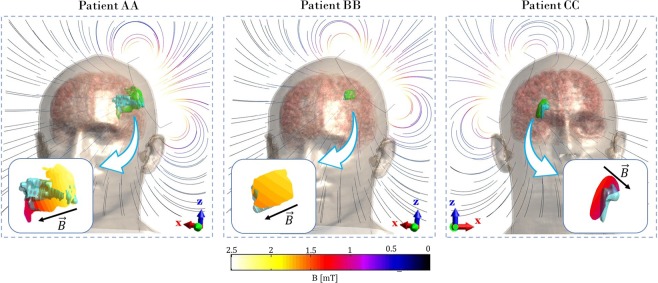
Streamline view of **B** field along the frontal plane (f-plane in the coil based coordinate, see Methods) for the three patients shows direction of the B field inside the head. In green: pre-treatment ischemic volume; in cyan: post-treatment ischemic volume. Blue inset shows the spatial gradient of ||**B**|| which the ischemia is exposed to during the treatment.

**Figure 2:**
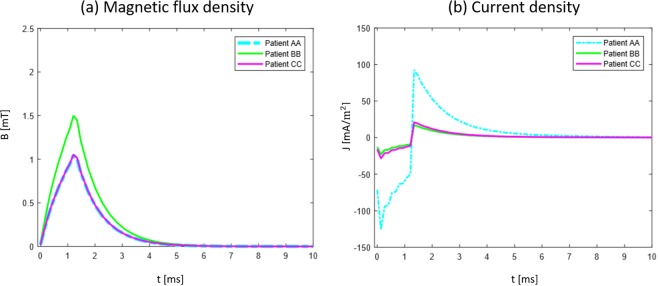
(**a**) Time course of ||**B**|| field for all patients in the voxel of the pre-treatment ischemic volume that experiences the lowest B. All the three patients undergo to at least 1 mT; (**b**) Time course of induced **J** for all patients in the voxel of the pre-treatment ischemic volume that experiences the highest value of **J**.

As expected, electromagnetic simulations show that the magnetic flux density field is not altered from the presence of the head, nor from each of its structures. In Fig. 1 streamline view of **B** field is shown together with the distribution of ||**B**|| over the surface of the ischemia (zoom in the inset) for each of the three patients. Data is shown through a color scale from black to white: intensities above 1 mT are represented as shading from red to white, while lower intensities ranges from purple to black. Here the gradient that the field experiences is clearly visible and its intensity is inversely proportional to the distance from the stimulating coil. *Patient AA* has the biggest lesion, which is exposed to a **B** field that varies from 1 mT to 2.2 mT, while those of *patient BB* and *patient CC* are smaller, so experiencing a more uniform **B** field. Between the two, being the ischemia of *patient BB* closer to the coil, it ensures an exposure to higher values (1.5 mT to 1.9 mT) with respect to that of *patient CC* (1 mT to 1.3 mT). Thus, the field that the lesion is exposed to depends on its dimension and position: the smaller the ischemia, the smaller the space variation of **B** values it experiences; the deeper, the lower is the intensity. In order to obtain the most reliable values of the **B** field and of the current density **J** induced in each voxel belonging to the ischemic volume we reconstructed the time course of the signal following the same approach to the electromagnetic solution as the one followed in Paffi *et al*.^22^, where a total of 150 simulations were performed. Fig. 2 reports the time course of ||**B**|| in the voxel that experiences the lowest ||**B**|| (Fig. 2a), and the time course of **J** inside the voxel that experiences the highest **J** (Fig. 2b). The most important observation is that all the three lesions are exposed to a field greater than or equal to 1 mT, which is the threshold of attested biological effects^13,14^. *Patient AA* and *patient CC* experience the same minimum value. This is because they both expand towards deeper regions of the brain. Regarding the time course of ||**B**|| in the voxel that experiences the maximum ||**B**|| field (Supplementary Fig. S1), the peak is slightly above 2 mT for *patient AA*, slightly below 2 mT for *patient BB* and slightly above 1 mT for *patient CC*. For the time course of **J** (Fig. 2b), *patient AA* is characterized by highest values due to the combined effect of the dimensions of the ischemia and the highest values of ||**B**|| (just above 2 mT). As a matter of fact, we expect a greater induction in a wider surface, according to Faraday’s law. For *patient BB* and *patient CC*, the different cross-sections of the two lesions force the two current densities to have similar values, despite experiencing different **B** field intensities. The absolute value of the negative peak of **J** is above 100 mA/m^2^ for *patient AA*, and above 20 mA/m^2^ for *patient BB* and *CC*.

Robustness of the semi-specific model with respect to changes in the anatomy of the head have been proven by simulating the ischemic volume inside two other generic head models: the Glenn model^34,35^ (ViP, cV3.1, 84-year-old man) and the MIDA model^36^ (28-year-old woman). For each model, the peak of the histogram that shows frequency of occurrence of B field intensities inside the ischemic volume was 2.01 mT and 1.95 mT, respectively, leading to a 5% maximum variation over the value computed with the Duke model (i.e. 2.05 mT), as shown in Supplementary Fig. S2.

**Figure 3:**
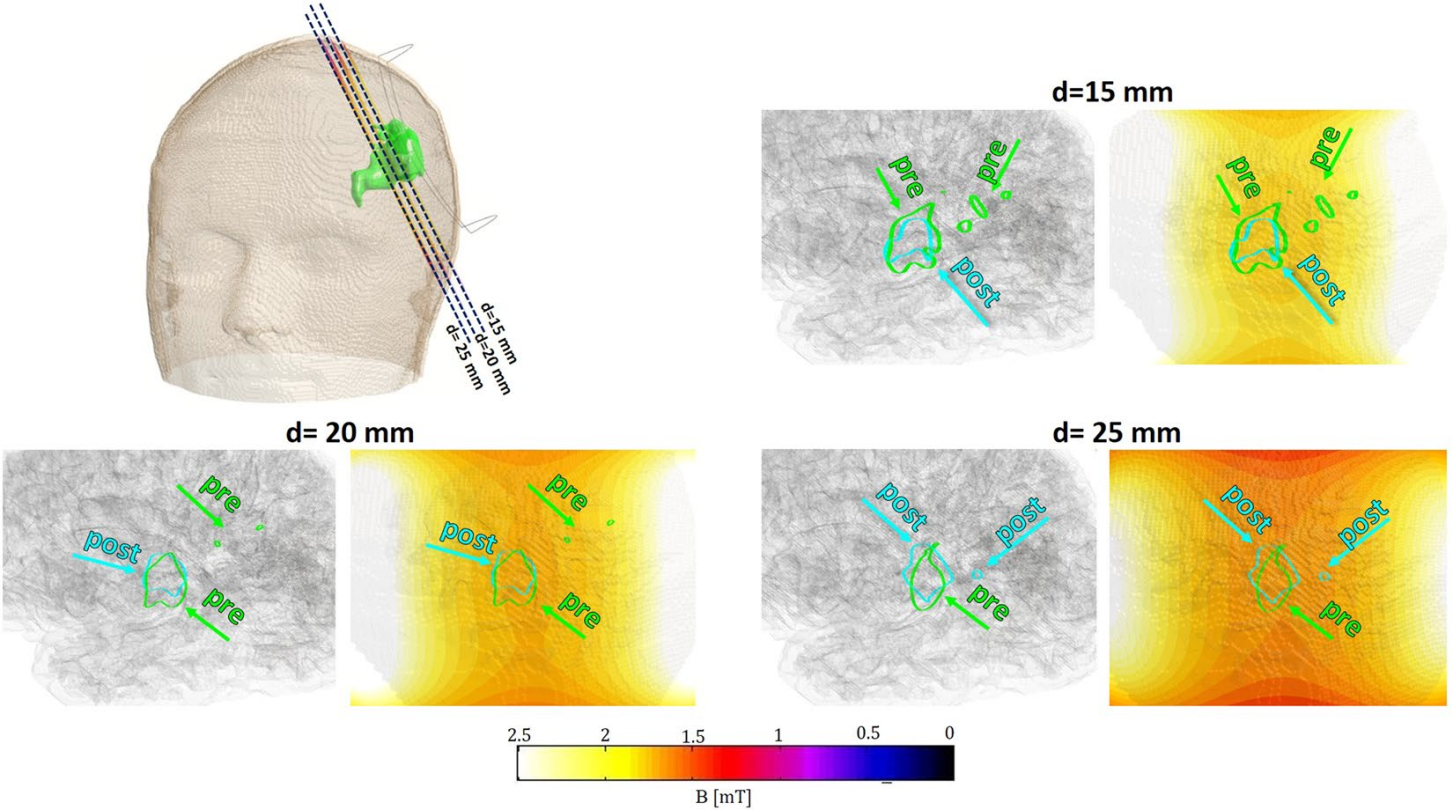
||**B**|| field distribution of *patient AA* on the planes parallel to the coil. Analysis is carried out at different distances *d* from the coil. For each distance, the edges of the slice of ischemia before (pre, in light green) and after (post, in cyan) the treatment are given, in order to compare the dimensions.

To better understand what the exposure level meant in terms of a possible reduction or containment of the growth of the ischemic volume, we superimposed the 3D model of the one month post-treatment ischemia to the pre-treatment one, as shown in Fig. 1 (cyan colored). We conducted an analysis comparing slices of the two volumes in the coil-based coordinate system (see Methods). As a representative example, results for *patient AA* are shown in Fig. 3 on planes parallel to the coil (*d-plane*, Methods). In this figure, the contours of pre- and post-treatment slices of ischemia are shown colored in light green and cyan respectively, at three different distances d from the coil. For each distance, left-side figures represent the slices inside the brain, for immediate inspection of the two contours. Distribution of ||**B**|| is shown in the right-side figures. From Fig. 3 one can observe higher post-treatment regions reductions with respect to pre-treatment ones with increasing intensity of **B** field. Similar analysis was conducted considering the distribution of ||**J**|| intensities. Results for *patient AA* on *d-plane* and *f-plane* are shown in Supplementary Figs. S3 and S4.

The data represented in Fig. 3 allows us to hypothesize that the volume reduction is an increasing function of the intensity of the fields experienced within the ischemic volume. In principle, this will permit the identification of a dose-response curve under proper conditions. Therefore, the following analysis was conducted in order to assess the best approach to attain such a result. Since the lesions are exposed to intensities greater than 1 mT, this is taken as the minimum exposure value. At first we chose 4 ranges for the **B** field and 5 for the **J** field and we quantified the partial volume of ischemia that is within each range, both for the pre-treatment ischemic lesion (*V*_*pre*_) and for the post- one (*V*_*post*_). Table 1 reports, for each exposure range, the lesion variations quantified as the ratio between *V*_*post*_ and *V*_*pre*_. Thus, a value above 1 indicates that the ischemic volume has enlarged, while a value below 1 shows that there’s been a reduction of the volume. Looking at the **B** field for *patient AA* and *patient BB*, we can see a visible reduction of the ischemic lesion (over 50%) when the exposure level is greater than 1.7 mT. However, when it comes to the regions exposed to less than 1.7 mT, data for *patient AA* show a two fold increase of the volume after the treatment, a value that is closer to the volume increase typical of untreated lesions. The same cannot be said for *patient BB* or *CC*, for whom the respective volumes exposed to the range from 1.3 mT to 1.7 mT (upper limit excluded) or from 1 mT to 1.3 mT (upper limit excluded) have slightly reduced after the treatment (up tp 12%). When considering **J**, each ischemic volume exposed to intensities above 5 mA/m^2^ has reduced for all the three patients (see Supplementary Table S1).

**Table 1:** Ratio between post-treatment and pre-treatment ischemic volume regions, selected based on the different ranges of exposure they experience.

	Ranges	Patient AA	Patient BB	Patient CC
		V_*post*_/V_*pre*_	V_*post*_/V_*pre*_	V_*post*_/V_*pre*_
B (mT)	1 ÷ 1.3	2.33	/	0.93
	1.3 ÷ 1.7	2.41	0.88	0.00
	1.7 ÷ 2	0.47	0.33	/
	≥2	0.14	/	/

The slash symbol shows that neither pre-treatment nor post-treatment ischemia is present in that specific region.

Such data confirm that it is possible to assess a dose response behavior but, due to intrinsic biological and patient variability, all the data needs to be pooled together in order to attain statistical significance. To move forward in this direction, we conducted a similar analysis looking at volumes defined only by lower limit thresholds instead of exposure ranges, see Methods for a detailed explanation.

**Figure 4:**
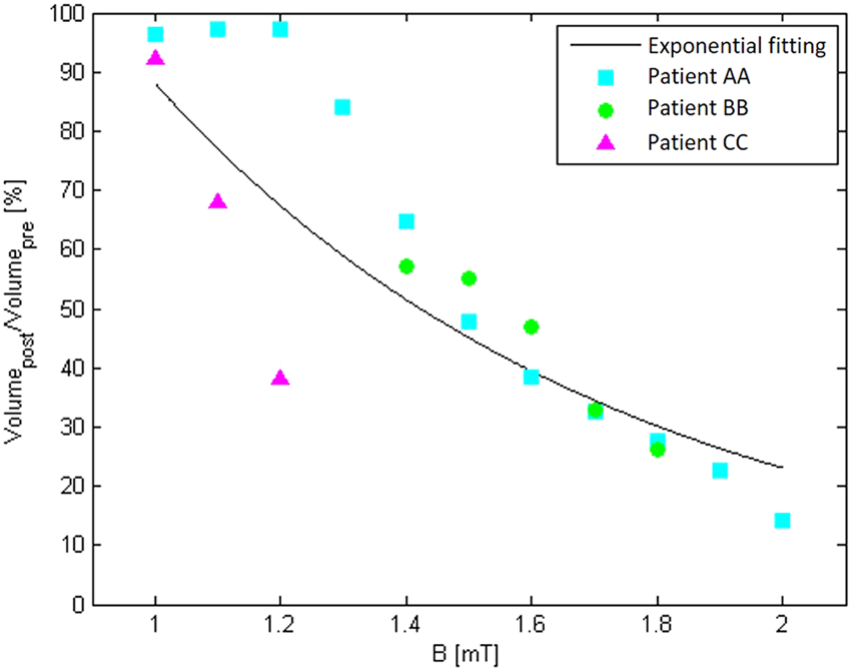
Scatter plot of the percentage quantity “ratio” with respect to increasing values of lower bound B field thresholds. Data for the three patients are pooled together, resulting in a decaying exponential function. Ratio is defined as the percentage of Volume_*post*_/Volume_*pre*_, where: Volume_*pre*_ = Ischemic pre-treatment volume that was exposed to B intensity greater than or equal to a certain threshold during the treatment. Volume_*post*_ = Ischemic post-treatment volume that is present in a region of the brain that was exposed to a B intensity greater than or equal to a certain threshold, during the treatment.

Fig. 4 shows the ratio between post-treatment and pre-treatment volumes as a function of ||**B**|| thresholds for all the three patients. The ratio is expressed as a percentage, so a value equal to 100% allows us to say that no reduction was obtained for that specific intensity. Because all the markers are below 100%, we can say that this treatment led to an overall reduction of the ischemic volume in the three patients considered. This analysis quantitatively confirms what previously inferred; each patient is characterized by different ratios but we can generally say that the higher is the intensity of exposure, the most effective is the treatment (i.e. the lower is the percentage of the post-treatment volume). Fig. 4 also shows an exponential fitting for data points of all patients pooled together; we show that a decaying exponential function well fits the values of this reduction. The function we used for the fitting is defined as $f(x)=M{e}^{ax}$, with x = B and *M* and *a* respectively equal to 79.38 and −0.1. The same analysis was conducted over the current density **J**, where a decaying exponential trend was found as well (See Supplementary Fig. S5).

## Discussion and Conclusions

In the context of biomedical applications based on electromagnetic stimulation, computational dosimetry is a fundamental tool to quantify the induced electromagnetic quantities related to the dose necessary to exert the effect. This is extremely important for the optimization of the application and to deepen the underlying mechanisms. Particularly, in the framework of stroke studies, some papers investigated how the presence of a lesion can alter the distribution of induced electric field and current inside the brain. Wagner and co-workers^37^ followed a stroke in its chronic state to understand the effects that electrical and anatomical changes can have on the current induced in the brain after exposure to TMS. To do so, they considered a numerical human healthy head model and 11 stroke models of different sizes and geometries. The stroke models were implemented by altering the cortical geometry and properties of the healthy head model guided by brain MRIs of patients with cortical strokes. They demonstrated that a stroke can drastically affect TMS application, by altering the location of the maximum current density and modifying the focus of the stimulation. A similar approach was adopted to estimate the electric field distribution inside brain stroke patients receiving tDCS (transcranial direct current stimulation)^38^. Here a generic spherical volume was placed inside the head of the human body models Ella and Glenn (Virtual Population - ViP^34,35^), to simulate an ischemic lesion. Additionally, electromagnetic properties of the sphere were varied to take into account both acute and chronic phase of the stroke. Similar to what was found for the TMS, results in Manoli *et al*.^38^ showed that the presence and type of lesion affected the maximum value and the distribution of the field induced in the brain after exposure to tDCS. Moreover, Manoli and co-workers^38^ demonstrated that patients in chronic phase benefit more from tDCS than patients in acute phase, given the higher contrast in tissue conductivity. The limitation of both these studies is that they don’t consider a realistic shape of the stroke, which becomes crucial when the dosimetric analysis is required to support a dose-response study on patient specific clinical treatment.

Specifically, in the context of ongoing studies intended to demonstrate whether PEMFs are able to elicit a neuroprotective effect on patients with ischemic stroke, the objective of the present paper was to identify a methodology for a dose-response curve assessment suitable to be applied in a large scale clinical study. To this scope, we analysed in details the exposure undergone in the study of Capone *et al*.^17^. In particular, we aimed to demonstrate that the type of exposure obtained with a single solenoid placed laterally to the head and co-centered with the ischemic lesion is able to induce a magnetic pulse with peak values above 1 mT under proper conditions. This intensity has been shown to exert effects on neuronal cells at the molecular level, increasing the density of adenosine receptors *A*_2*A*_ and *A*_3*A*_ on the cell membrane^9,10^. To verify the intensity of **B** field which each patient has been exposed to during the treatment, electromagnetic simulations were performed on a patient semi-specific head model. Such model was realized using the geometric information of the ischemic area, obtained from the MRI data of the patient, that had been then placed inside a standard computational model. Results of the simulations showed that the ischemic volumes in the brain experienced a magnetic flux density field always above the desired 1 mT. Moreover, quantitative post-elaboration studies were performed on the ischemic lesion. Particularly, while the analysis of ischemic slices qualitatively suggested us a correlation between ischemic lesion reduction and **B** field values experienced, the volumetric analysis allowed us to assess the percentage of volume associated to specific thresholds. In this sense, the fitting curve of Fig. 4 takes into account post- and pre-treatment volumes ratio above a certain given lower field threshold, showing that increasing such a value (i.e. excluding volumes experiencing lower fields) decreases the ratio, ultimately indicating a better efficiency in eliciting the effect. As an example, by looking at Fig. 4 we can say that a 50% reduction of the volume was obtained by exposing to a **B** field of intensity around 1.4 mT, or, similarly, by inducing a current density of about 5 mA/m^2^ (Supplemetary Fig. S5). Fig. 4 can be considered the example on how to attain a dose response curve when a sample of volunteers large enough will be used.

In conclusion, we have demonstrated that, by using electromagnetic simulations on virtual body models combined with patient specific lesions, it is possible to build a dose–response curve in an “*electroceutical*” perspective^19^ when dealing with a large number of patients. This is necessary if one, aiming to introduce a new electromagnetic–based therapy, like the I-NIC, wants to follow an approach similar to the drugs clinical trials, as an example one can think to a typical *Phase*2 study^39^. *Limitations*: This methodology was conceived for the ongoing I-NIC study^18^, but here it is applied to the reduced sample of the three patients recruited in Capone *et al*.^17^ that underwent magnetic stimulation for 120 minutes (the same treatment duration as the one used in the I-NIC study^18^). Thus, such an approach will be a powerful tool to a reliable assessment of a dose-response threshold of the treatment when a large number of patients will be evaluated.

## Methods

The study complied with the Helsinki declaration and was approved by the local ethics committee (Università Campus Bio-Medico di Roma) and Italian Ministry of Health. Informed consent was obtained from all participants. The study was registered with ClinicalTrials.gov, number NCT01941147 (September 9, 2013). The study protocol has been published^40^. Numerical dosimetry was performed on the three subjects exposed for 120 minutes.

**Figure 5:**
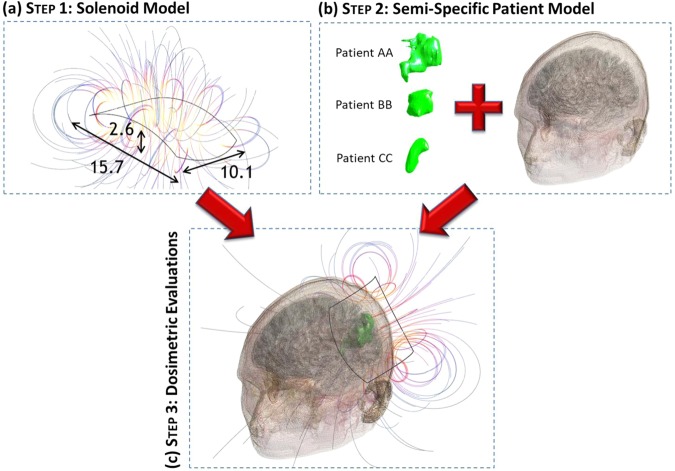
Sketch of workflow, (**a**) step 1: solenoid model (experimentally validated), (**b**) step 2: semi-specific model based on reconstructed ischemic volume for each patient, (**c**) step 3: dosimetric evaluations.

To perform the dosimetric analysis, we used the software Sim4Life v.3.4 (ZMT, Zurich MedTech AG), with the Magnetic Quasi Static module. The model used to perform the EM simulations has been defined as semi-specific, this because the 3D model of the ischemic lesion of each patient that underwent the 120-minutes-treatment was entered in a standard human head model, obtained from the Virtual Population member Duke (v.1.0, Zurich MedTech AG^34^). This version of Duke is a 1 mm voxeled male model counting a total number of 77 different tissues, of which we have selected only the 40 structures of the head. Electric properties of tissues are built-in in Sim4Life v.3.4 and they are based on IT’IS database^41^. In order to perform the EM simulation, computational model of the stimulation device must be considered as well. The concept behind the semi-specific model and the final geometry are shown in Fig. 5. A detailed description is reported as follows.

### Coil model and simulation parameters

**Figure 6:**
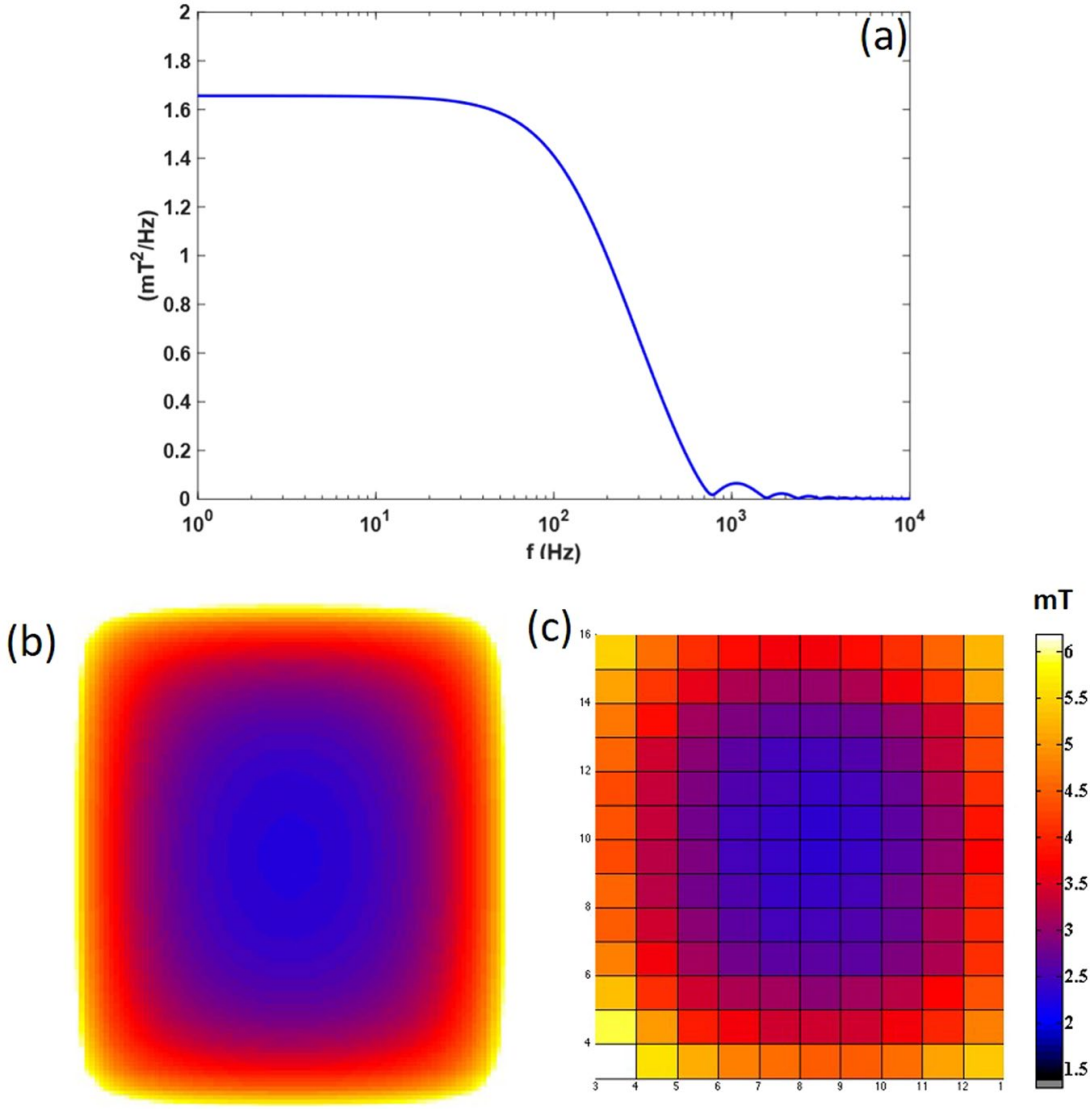
Stimulation model. (**a**) Pulsed signal in frequency domain; simulated ||**B**|| (**c**) and measured ||**B**|| (**d**) on the plane of the coil. Measurements are conducted with the Gaussmeter/Teslameter Model DG-50, from the company Laboratorio Elettrofisico.

The medical device used by IGEA company for both the study conducted in Capone *et al*.^17^ and in the I-NIC clinical study^18^ to deliver PEMFs consists in a generator of a pulsed current signal that supplies an external coil (solenoid), that, in turn, generates a pulsed electromagnetic field, with a duration of the active phase of the signal equal to 1.3 ± 0.1 ms and a repetition frequency of 75 Hz^16^. The coil is a 240-turned solenoid with a rectangular shape and is held in position upon the ischemic hemisphere with a helmet, placed on the head of the patient by means of a Velcro strap^17^. This geometry was reproduced in the simulation environment, where a single turn rectangular coil of 14 × 10.6 cm (mean length of the 240 turns of the solenoid), with no thickness, was designed. The peak value of the pulsed current signal that feeds the rectangular coil is 240 A. The solenoid model was validated after comparison between measured and simulated B field distribution over the coil plane at the time peak (Fig. 6b,c). After validation, for the three different patient conditions, the coil, was properly warped in order to simulate the solenoid properties of being adaptable to the head shape (Fig. 5a).

As it’s not possible to work in time domain when using Sim4Life v.3.4.0, authors followed the same procedure described in Paffi *et al*.^22^, that has been implemented based on the harmonic decomposition of the pulsed signal simulations. This method allows accounting for frequency dependence of both conductivity and permittivity of brain tissues in the signal frequency band. Therefore, 150 simulations were conducted in the frequency range [50–7500] Hz with a step of 50 Hz. The aforementioned range contains the frequency components necessary to appropriately reconstruct the time course of the pulsed current signal, whose Power Spectral Density is reported in Fig. 6a. Time course of magnetic flux induction B and current density J induced in the ischemic tissue was previously described in Fig. 2 of the Results.

### Ischemic volumes: from MR images to 3D model

The 3D model of the ischemic tissue was obtained by segmenting the MRI scans of each of the patient that underwent the treatment for 120 minutes. MRI data is shown in grey scale, giving the possibility to distinguish different tissues. Particularly, the ischemic lesion is clearly visible, among all the other brain structures, as it is strongly hyperintense. The procedure adopted to obtain the 3D model of the ischemia has been described in Capone *et al*.^17^, and will be briefly summarized here:

1. Lesion area is segmented with a semi-automatic region growing segmentation tool, followed by a manual editing of the edges.
2. After the segmentation, the voxels belonging to the ischemic volume are marked with a unitary value, while all the others are considered equal to zero. In this way, we obtain a binary mask that contains only the geometry of the ischemia.
3. After conversion in a .raw file, each binary mask is imported in Sim4Life v.3.4 and processed in order to generate the surface of the ischemia. Since the resolution of these data, especially in the longitudinal axis, is lower than the one of the head model, a smoothing function with an iterative algorithm that adapts the two different discretizations have to be applied.

This procedure was implemented to obtain, for each patient, two different models of the ischemia: before and one month after the treatment. In Fig. 5b, the model of each pre-treatment ischemia is reported in green. The model representing the pre-treatment volume was included in the dosimetric study, as it simulates the lesion treated with the PEMFs stimulation. Instead, post-treatment ischemia is considered only during post-processing to evaluate and quantify variations of the geometry, in terms of area and volume.

### Assembly of the model and dosimetric problem solving

**Figure 7:**
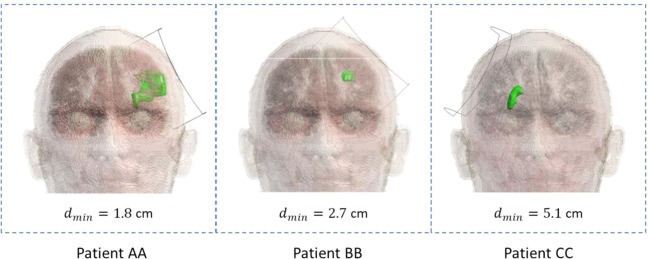
Final semi-specific patient models: all patients are shown on xz-plane. The shortest distance between the ischemia and the coil is defined as *d*_*min*_.

Once both the coil and the ischemia models are created, they must be conveniently placed with respect to Duke’s head, following the idea described in Fig. 5. First of all, it was necessary to put the ischemic volume inside the head model. A first placement was obtained by importing MRI slices of the whole head of each patient in Sim4Life and adjusted to those of Duke, in order to find landmarks that helped for a first positioning of the ischemia. Final adjustment was obtained by visual inspection with physicians. After this, the coil was placed as close as possible to the head, with the ischemic volume centered along the coil axis. In this way, we obtained three different configurations corresponding to the three different patient conditions, as shown in Fig. 7. Additionally, to evaluate the effect of a possible misplacing of the stimulator during the treatment, the coil was shifted by ±1 cm over the head along the longitudinal and transversal directions in patient AA. Results showed a 2% maximum variation for the peak of the histogram that shows frequency of occurrence of B field intensities inside the ischemic lesion (see Supplementary Fig. S6), demonstrating robustness over changes in the position of the coil. As already discussed above, we solved the electromagnetic problem considering only the pre treatment ischemia, to which electric properties of the edema^42^ were assigned, which means a conductivity *σ* equal to 1.7 S/m and the same relative permittivity ${\varepsilon }_{r}$ as the grey matter. Head tissues were automatically assigned through IT’IS database. To perform the simulation, a 0.5 mm and a 1 mm isotropic resolution were imposed to the ischemia and to Duke’s skin respectively, whereas Sim4Life default resolution of 0.625 × 0.625 × 0.625 mm was imposed to the remaining structures.

### Comparison between pre-treatment and post-treatment volumes

**Figure 8:**
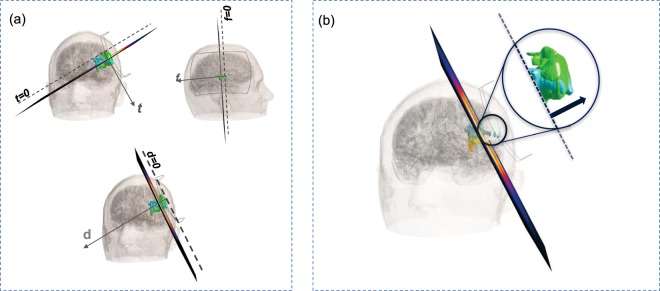
(**a**) Planes relative to the coil used to observe pre-treatment and post-treatment ischemic slices. (**b**) Example of volume selection: here the threshold is set for B to 1.7 mT. Inside the blue smaller circle: the volume of ischemia exposed to a B field equal or greater than 1.7 mT is represented in white; how that partial volume has changed after the treatment is shown in cyan.

Aiming to obtain a correlation between intensity of the electromagnetic quantities (**B** and **J**) and changes in ischemic volume and geometry, a first surface-based analysis was conducted on single slices of the lesion. In this sense, the 3D models of pre-treatment and post-treatment ischemia were cut in slices along specific directions, with a relative distance between two consecutive slices equal to 5 mm. In order to conduct this study, we chose to work with a coil-based frame of reference, instead of the default cartesian coordinate system X-Y-Z. The new system consists of three orthogonal axis, defined as *d*, *f* and *t*, where *d* is the normal to the *d-plane*, which is parallel to the coil, as it passes through its four vertices. While axes *t* and *f* are defined as the vectors normal to the *t-plane* and the *f-plane*, respectively. These two planes are orthogonal to the coil, because they pass through two of its four vertices, i.e. chosen as those belonging to the short or the long side of the coil, respectively. The three planes described above are shown in Fig. 8a, where it is also shown that each plane is shifted along its normal, in order to obtain the slicing of both pre-treatment (green) and post-treatment (cyan) lesion. With this procedure, we did a first evaluation of pre- and post-treatment regions, as extensively described in the results. However, this surface-based analysis is limited by the finite dimensions of the ischemia, which could cause a misreading of data for those slices bordering with the extremities of the object. For this reason, rather than performing a quantitative evaluation with this procedure, we decided to focus on volumetric considerations. We applied the same concept when considering both the magnetic flux density field, **B**, and the induced current density, **J**. Either way, we selected as thresholds specific intensities within the range of all the values experienced by the ischemic volume. The amount of pre-treatment volume exposed to a field equal or greater than the selected threshold was computed. In order to apply this evaluation to the post-treatment ischemia, we identified exposure levels on brain regions where the ischemic volume is present one month after the treatment. Thus, considering the same thresholds, we could compute the amount of post-treatment ischemic volume that lies on regions previously exposed to the given field values. The purpose of this analysis was to assess how much post-treatment volume persisted in a region treated with intensities at least equal to the chosen threshold. In Fig. 8b we visually describe how this procedure works when considering the **B** field: as already mentioned in the Results, **B** is homogeneously distributed along the *d-plane* and its intensity depends only on the distance from the coil. For this reason, post- and pre-treatment ischemic volume exposed to a magnetic field equal or greater than the set threshold will be approximately all above the plane parallel to the coil, placed at a specific distance *d* from it. However, this simplified representation is only possible when considering the **B** field, while the distribution of **J** is strongly influenced by the encountered tissues.

## Supplementary information

Supplementary Information.

## Data Availability

Data generated and/or analyzed during the current study are available from the corresponding author on reasonable request.
