# Supplementary material for: Patient Semi-specific Computational Modeling of Electromagnetic Stimulation Applied to Neuroprotective Treatments in Acute Ischemic Stroke
Source: Sci Rep. 2020 Feb 19;10:2945. doi: 10.1038/s41598-020-59471-9 (PMC7031527; doi:10.1038/s41598-020-59471-9)
Supplement: Supplementary file 1 — Supplementary Information. [file 41598_2020_59471_MOESM1_ESM.docx]

# **Supplementary Information**

# **Patient Semi-specific Computational Modeling of Electromagnetic Stimulation Applied to Neuroprotective Treatments in Acute Ischemic Stroke**

Micol Colella^1,+^, Francesca Camera^1,+^, Fioravante Capone^2^, Stefania Setti^3^, Ruggero Cadossi^3^, Vincenzo di Lazzaro^2^, Francesca Apollonio^1^ and Micaela Liberti^1,*^

^1^Department of Information Engineering, Electronics and Telecommunication (DIET), University of Rome, “La Sapienza”, Rome, Italy.

^2^Unit of Neurology, Neurophysiology, Neurobiology, Department of Medicine, Università Campus Bio-Medico di Roma, Rome, Italy.

^3^IGEA Biophysics Laboratory, Carpi, Italy.

^*^micaela.liberti@uniroma1.it.

^+^these authors contributed equally to this work.

**Time course of B field**
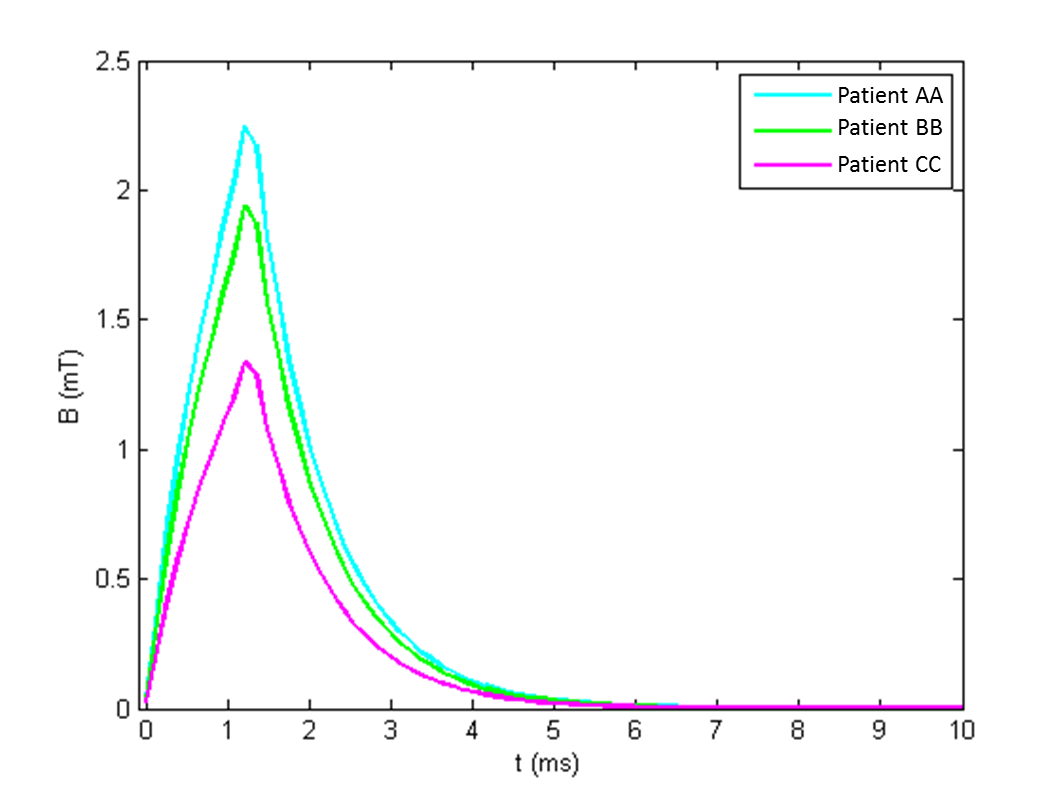


**Supplementary Figure S1.** Reconstructed time course of B field for all patients in the voxel of the pre‑treatment ischemic volume that experiences the highest B. The aforementioned voxel is the one closest to the coil.

**Frequency of occurrence of |B| inside the ischemic volume for different head models**

Histograms in Figure S2 show frequency of occurrence of B field intensities inside the ischemic volume, for three different human head models. Peak of each histogram is 2.05 mT for the Duke model, 2.01 mT for the Glenn model and 1.95 mT for the MIDA model. Thus, maximum variation of the peak is 5 %.


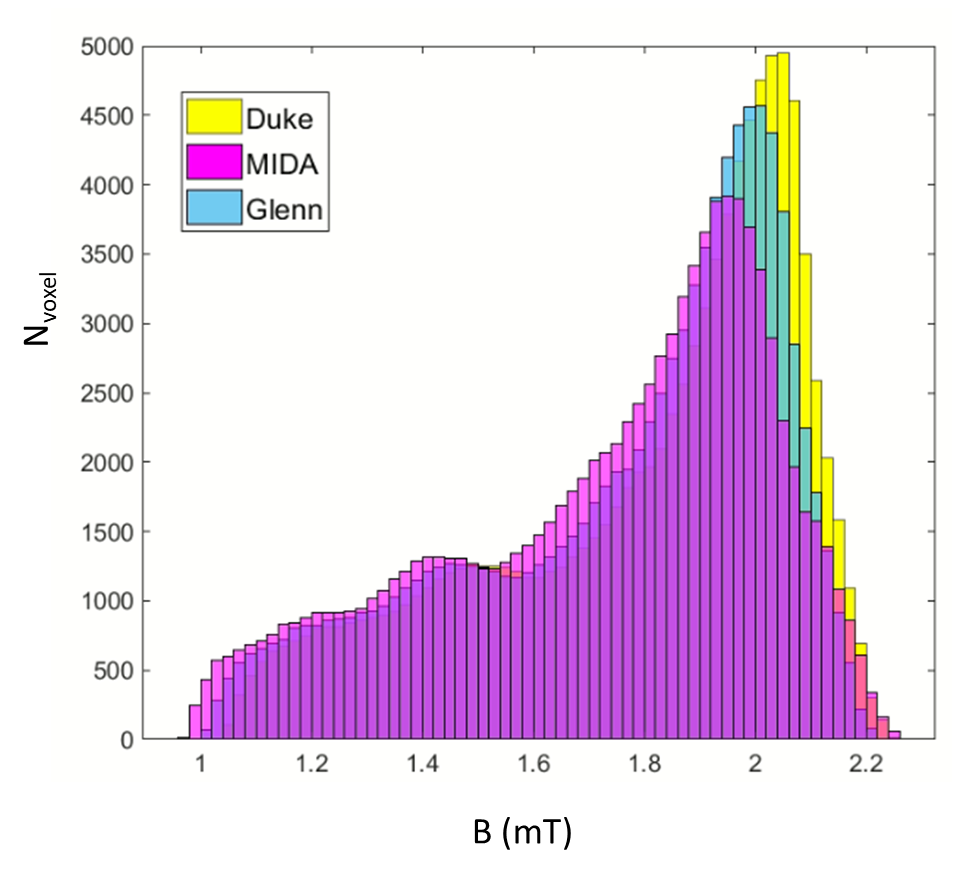


**Supplementary Figure S2.** Histograms showing frequency of occurrence of B inside the ischemic volume when placed in three different human head models, i.e. Duke, Glenn and MIDA.

**Intensity distributions for the current density J**

Following results were obtained from simulation conducted at 250 Hz. This frequency is chosen as the frequency that best approximate J(t) at the first peak time.


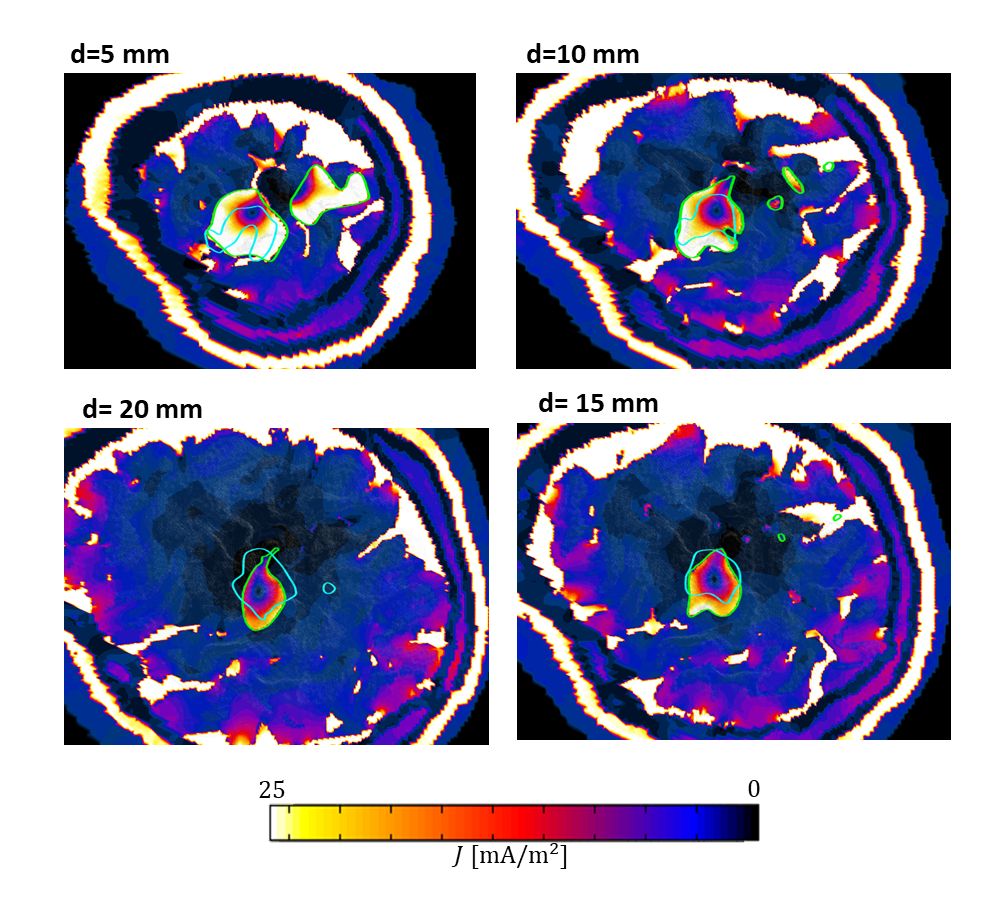


**Supplementary Figure S3.** Distribution of |**J**| on the plane parallel to the coil, for patient AA. Analysis is carried out at different distances ‘d’ from the coil. For each distance, the edges of the slice of ischemia before (pre, in light green) and after (post, in cyan) the treatment are given, in order to compare the dimensions.


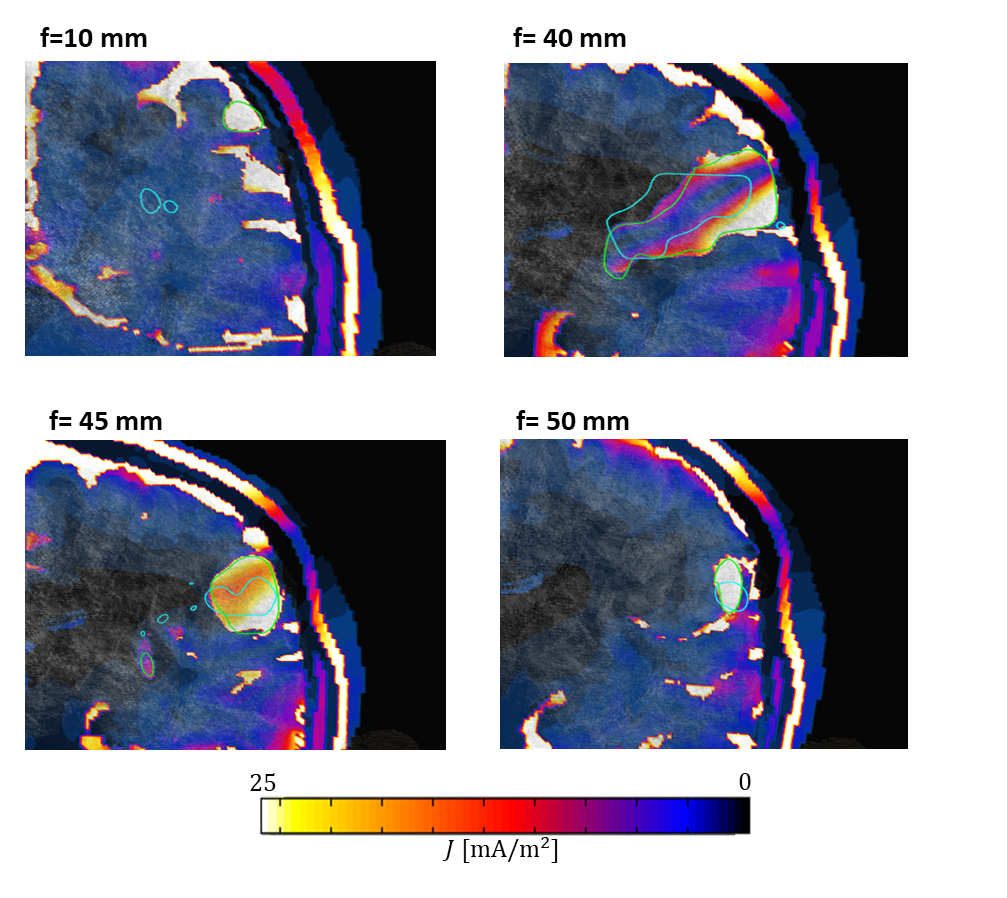


**Supplementary Figure S4.** Distribution of |**J**| on the frontal plane orthogonal to the coil (f-plane), for patient AA. Analysis is carried out at different distances ‘f’ from the coil. For each distance, the edges of the slice of ischemia before (pre, in light green) and after (post, in cyan) the treatment are given, in order to compare the dimensions.

**V*_post_/*V*_pre_* Ratio: exposure classification**

Lesion variations are quantified as the ratio between V_post_ and V_pre_ and evaluated for different exposure ranges.

**Supplementary Table S1.** Ratio between post‑treatment and pre‑treatment ischemic region of volumes, selected based on the different ranges of exposure they experience. The slash symbol shows that neither pre‑treatment nor post‑treatment ischemia is present in that specific region.

|  | **Ranges** | **Patient A**  V*_post_/*V*_pre_* | **Patient B**  V*_post_/*V*_pre_* | **Patient C**  V*_post_/*V*_pre_* |
| --- | --- | --- | --- | --- |
| **J (mA/m^2^)** | 1÷5 | 5.77 | 0.76 | 2.34 |
|  | 5÷15 | 0.64 | 0.42 | 0.51 |
|  | 15÷30 | 0.30 | 0.06 | 0.17 |
|  | 30÷45 | 0.16 | / | 0.00 |
|  | >45 | 0.05 | / | / |

**Dose-response curve using J data**

Ratio between post‑treatment and pre‑treatment volumes as a function of |**J|** thresholds, for all the three patients and exponential fitting for data points of all patients pooled together. The function used for the fitting is defined as f(x)=Me^ax^, with x = J and M and a respectively equal to 336.13 and -1.34.


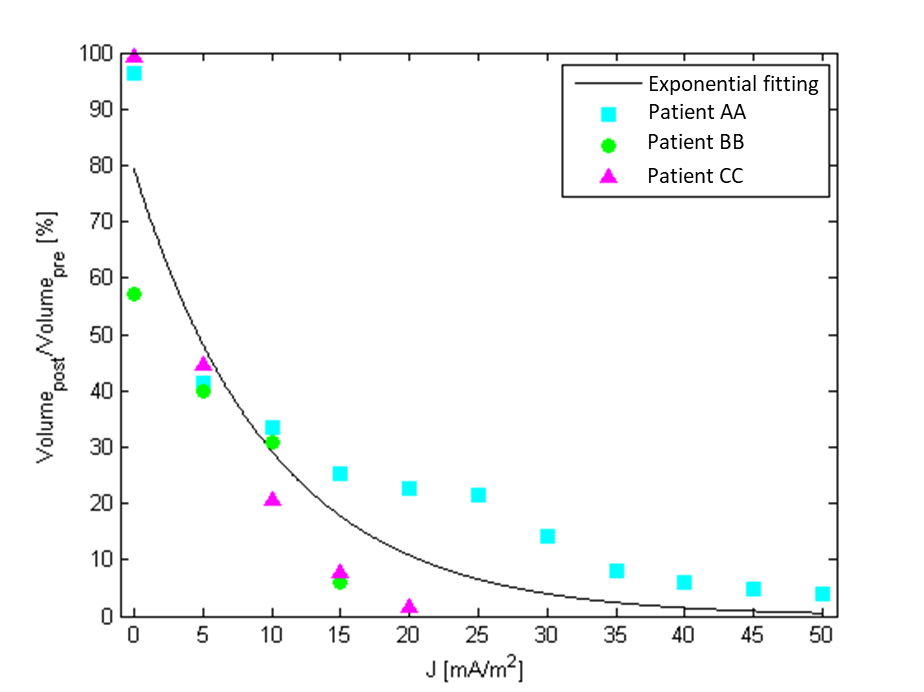


**Supplementary Figure S5.** Scatter plot of the percentage quantity "ratio" with respect to increasing values of lower bound J field thresholds. Data for the three patients are pooled together, resulting in a decaying exponential function. Ratio is defined as the percentage of Volume_post_/Volume_pre_, where:

Volume_pre_= Ischemic pre-treatment volume that was exposed to B or J intensity greater than or equal to a certain threshold during the treatment.

Volume_post_ = Ischemic post-treatment volume that is present in a region of the brain that was exposed to B or J intensity greater than or equal to a certain threshold, during the treatment.

**Frequency of occurrence of |B| inside the ischemic volume for different coil positions**

Histograms in Figure S6 show frequency of occurrence of B field intensities inside the ischemic volume, for different positions of the coil. The peak for each histogram ranges between 2.01 mT and 2.07 mT, thus maximum percentage variation is 2%.


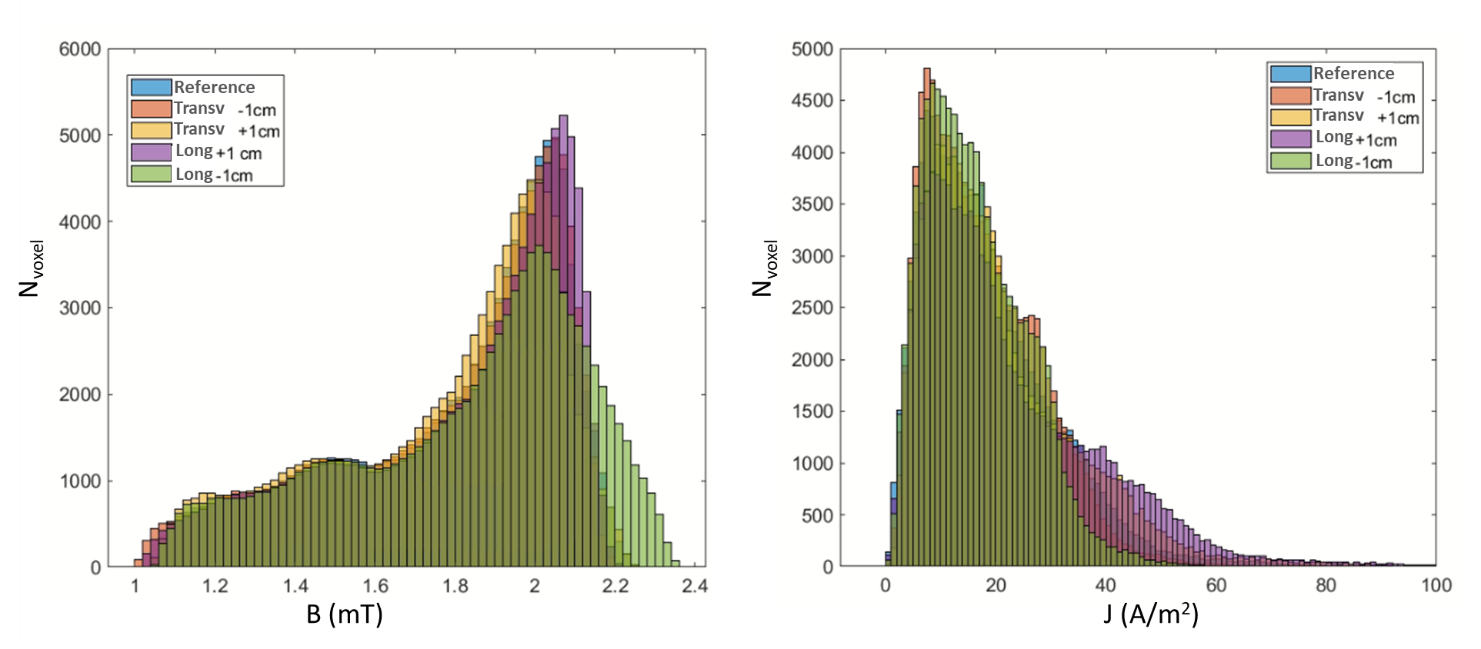


**Supplementary Figure S6.** Histograms showing frequency of occurrence of |**B**| inside the ischemic volume when placing the coil in different positions over the head. In the reference position, ischemic volume is centered along the coil axis. To explore other placements, the coil was shifted by ± 1 cm along the longitudinal axis and the transversal axis of Duke’s head.
